# Detecting the movement and spawning activity of bigheaded carps with environmental DNA

**DOI:** 10.1111/1755-0998.12533

**Published:** 2016-05-09

**Authors:** Richard A. Erickson, Christopher B. Rees, Alison A. Coulter, Christopher M. Merkes, Sunnie G. McCalla, Katherine F. Touzinsky, Liza Walleser, Reuben R. Goforth, Jon J. Amberg

**Affiliations:** ^1^ U.S. Geological Survey Upper Midwest Environmental Sciences Center La Crosse WI USA; ^2^ Department of Forestry and Natural Resources Purdue University West Lafayette IN USA; ^3^ Present address: US Fish and Wildlife Service Northeast Fishery Center Lamar PA USA; ^4^ Present address: Center for Fisheries, Aquaculture and Aquatic Science Southern Illinois University Carbondale IL USA; ^5^ Present address: Wisconsin Department of Natural Resources Rhinelander WI USA

**Keywords:** Asian carp, bigheaded carp, environmental monitoring, fisheries management, invasive species detection

## Abstract

Bigheaded carps are invasive fishes threatening to invade the Great Lakes basin and establish spawning populations, and have been monitored using environmental DNA (eDNA). Not only does eDNA hold potential for detecting the presence of species, but may also allow for quantitative comparisons like relative abundance of species across time or space. We examined the relationships among bigheaded carp movement, hydrography, spawning and eDNA on the Wabash River, IN, USA. We found positive relationships between eDNA and movement and eDNA and hydrography. We did not find a relationship between eDNA and spawning activity in the form of drifting eggs. Our first finding demonstrates how eDNA may be used to monitor species abundance, whereas our second finding illustrates the need for additional research into eDNA methodologies. Current applications of eDNA are widespread, but the relatively new technology requires further refinement.

## Introduction

Invasive species adversely affect both ecosystem functions and economic activities by outcompeting native species, degrading habitats and decreasing the quality of recreational activities (Lockwood *et al*. 2007; Pejchar & Mooney 2009; Simberloff *et al*. 2013). Additionally, many invasive species are undesirable weeds that impede agriculture (Daehler 1998) and rangeland management (DiTomaso 2000). Overall, invasive species cause billions of dollars of damage and losses to the economy of the United States (Pimentel *et al*. 2000, 2005). Bigheaded carps (*Hypophthalmichthys* spp.), a subgroup of Asian carps, are invasive species that have spread across North America via the Mississippi River system (Kolar & Lodge 2002). These species can outcompete native fishes (Irons *et al*. 2007) and degrade habitat by changing both plankton communities (Sass *et al*. 2014) and aquatic vegetation (Buck *et al*. 2010). The indirect effects of these changes impact a wide range of native species, ranging from plankton to waterfowl (Buck *et al*. 2010). Bigheaded carps, including silver carp (*Hypophthalmichthys molitrix*), bighead carp (*Hypophthalmichthys nobilis*) and their associated hybrids, are currently threatening to invade the Laurentian Great Lakes and establish spawning populations (Kolar *et al*. 2007). Several potential routes have been identified by which bigheaded carp may invade the Great Lakes (US Army Corps of Engineers 2010), including via the Wabash and Maumee Rivers through Eagle Marsh near Fort Wayne, IN, USA (although Eagle Marsh is scheduled to be closed in spring 2016; Jeff Heath, USACE, personal communication). While steps have been taken to help maintain separation at many of these possible invasion routes, species invasions may still be possible.

Detecting and monitoring the spread of invasive species is critical for their management, and surveillance programmes have been initiated in both the United States and Canada (Cudmore *et al*. 2011; Jerde *et al*. 2013). These surveillance programmes guide management activities because control programmes require basic knowledge such as the occurrence and abundance of species to plan management actions. Traditionally, aquatic invasive species have been monitored using methods such as microscopy for early life stages [e.g. zebra mussel; Johnson (1995)], collecting oocytes [e.g. DeGrandchamp *et al*. (2007)], electroshocking [e.g. Wilson *et al*. (2014)], netting (Jerde *et al*. 2011), using Judas fish [e.g. Bajer *et al*. (2011)] and conducting mark–recapture studies [e.g. Sass *et al*. (2009)]. These methods require intensive personnel efforts that make their application costly. Furthermore, these methods have additional limitations. For example, netting and electroshocking can adversely affect nontarget species (Hoffman *et al*. 2011). Moreover, some invasive species are skilled at avoiding capture in nets; are sensitive to electrical fields and therefore able to avoid electroshocking; or are cryptic, small or similar in appearance to native species (Hoffman *et al*. 2011). Newer detection methods such as environmental DNA (eDNA) allow sampling to detect invasive species without many of these shortcomings (Lodge *et al*. 2012). In fact, a 2012 issue of *Molecular Ecology* focused on the use of eDNA (Lodge *et al*. 2012; Thomsen *et al*. 2012; Yoccoz 2012). Environmental DNA methods were initially developed to generate presence/absence data for species; however, recent work has demonstrated that the amount of DNA present may also be correlated with abundances, population sizes and biomass of aquatic species (Lodge *et al*. 2012; Takahara *et al*. 2012; Thomsen *et al*. 2012; Pilliod *et al*. 2013; Klymus *et al*. 2015). Known limitations exist such as a few large fish shedding a comparable amount of DNA as many small fish (Klymus *et al*. 2015).

The possible relationship between eDNA concentrations and other important life history events remains uninvestigated. In the case of bigheaded carps, knowledge of both seasonal movement and spawning activities are life history events that may be essential for control and management. Bigheaded carps have specific hydrologic conditions which must be met to induce spawning, and specific spatial and hydrologic conditions to allow egg and larval development. Both spawning and early development also require turbulent conditions until vertical swimming occurs. Specifically, spawning occurs on the rising limb of the hydrograph, at least in their native habitat on the Yangtze River, at turbulent riparian sites when appropriate water temperatures (>25 °C) are achieved (Yi *et al*. 1988; Li *et al*. 2006; Kolar *et al*. 2007). The same hydrological conditions that trigger spawning events also cause carp movements within the existing range of a species (Deters *et al*. 2012).

We sought to characterize the relationships among eDNA, hydrography, fish movement and spawning events by conducting a series of natural experiments in the Wabash River, IN, USA. The Wabash River is a tributary of the Ohio River, which flows into the Mississippi River. The headwaters of the Wabash River are intertwined with the Great Lakes watershed. Additionally, the river offers a gradient of carp densities, with fewer carps found upstream (Coulter 2015). We conducted traditional monitoring activities that included both drifting egg surveillance to indicate spawning activity and telemetry of acoustically tagged bigheaded carps at large in the river. We hypothesized that both bigheaded carp movement and spawning events would be correlated with eDNA levels and river discharge.

## Materials and methods

### Study sites and site selection

The Wabash River is a tributary of the Mississippi River that is connected to the Great Lakes watershed via Eagle Marsh (US Army Corps of Engineers 2010). Surveys of spawning activity were conducted on Wabash River between river kilometres (rkm) 480–640 during June–September 2011 and May–June 2012, and acoustically tagged fishes were monitored using both passive and active telemetry over the same spatiotemporal context (Coulter 2015).

**Figure 1:**
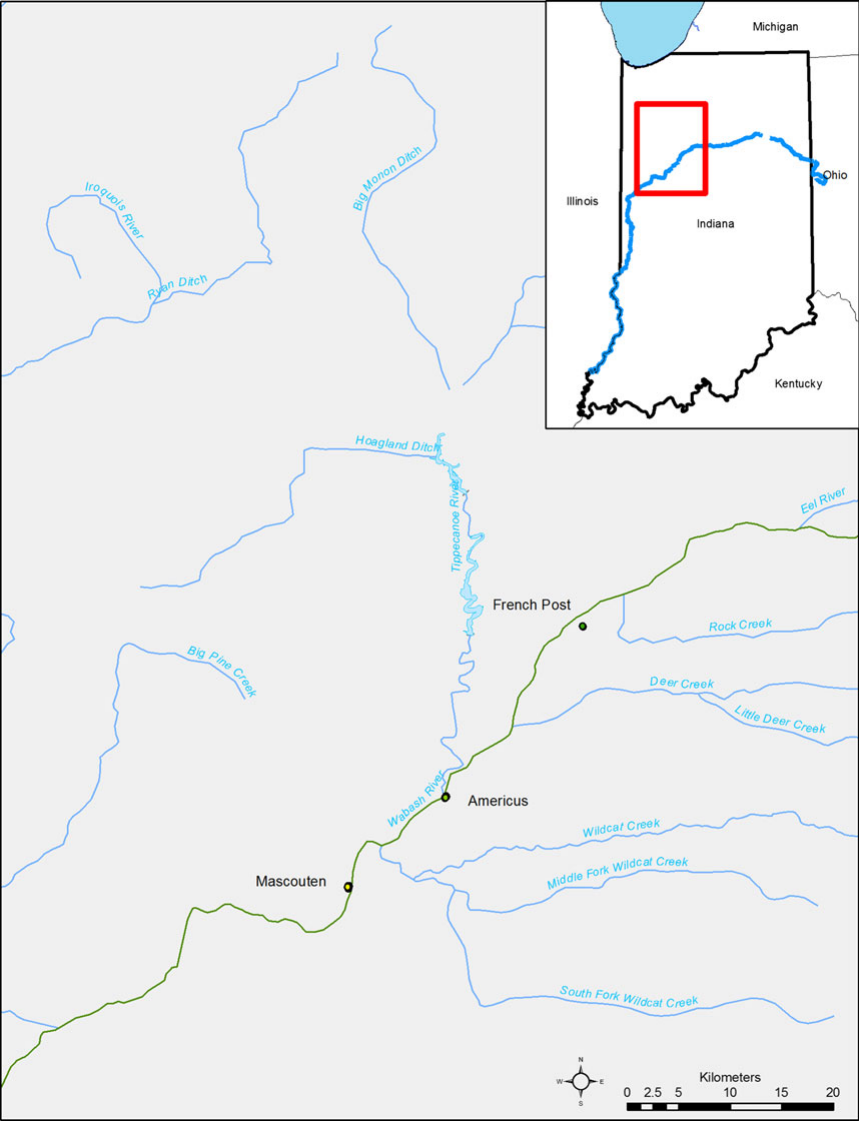
Study sites on the Wabash River, Indiana, USA (French Post, Americus and Mascouten). Open circles represent positions of stationary Vemco receivers for telemetry data collection. Site names are noted for each sampling location.

At three sites along the Wabash River (Fig. 1), we collected eggs, water samples for eDNA extraction and fish movement data (at one site) between May 29, 2013 and June 22, 2013. We selected the Mascouten Boat Launch (hereafter, Mascouten), located near West Lafayette, IN, at rkm 499, because it was at the centre of an ongoing telemetry study and bigheaded carp eggs had previously been collected at the location (Coulter *et al*. 2013; Lenaerts *et al*. 2015). Additionally, backwater habitat thought to be used by bigheaded carps for staging prior to movement to spawning locations runs is located immediately downstream of this site (Coulter *et al*. 2012), which may change eDNA levels as fish move to spawn. We selected Americus, located at the confluence of the Tippecanoe and Wabash Rivers at rkm 518, because it was near a stationary telemetry receiver and bigheaded carp eggs had previously been collected at the site (Coulter *et al*. 2013). We selected French Post, located at rkm 531, as the final site because relatively few bigheaded carps were found at the site in previous surveys (Coulter 2015).

### Hydrographic data

We downloaded daily discharge data from the U.S. Geological Survey (USGS) National Water Information System for the Wabash River at Lafayette, IN, USA (# 03335500), and at Logansport, IN, USA (# 03329000), for the time period covering our study dates: May 29, 2013 to June 22, 2013 (U.S. Geological Survey 2016). We used the Lafayette gage to calculate discharge at the Mascouten site. The USGS Illinois Water Sciences Center used the Logansport gage, which is located upstream of the Americus and French Post sites, to estimate the discharge values at both sites using the drainage area ratio method (Ponce 1994). This calculation allowed for discharge values at one site to be used to estimate discharge values at a second site by assuming the ratio of discharges at two locations is approximately equal to the ratio of the drainage area. French Post has a drainage area of 1 013 000 ha, Americus has a drainage area of 1 134 000 ha and Logansport has a drainage area of 979 000 ha.

### Telemetry

We captured and implanted 300 bigheaded carps, mostly silver carps, with acoustic telemetry tags from 2011 to 2013 at several locations along the Wabash River (Coulter 2015). Vemco (Vemco, Bedford, Nova Scotia, Canada) individually coded ultrasonic transmitters (Model V16‐4L, 24 g, 16‐mm diameter, 68‐mm length) were surgically implanted in the coelomic cavity of each fish. A stationary receiver (Vemco VR2Ws; Coulter 2015) deployed at rkm 521 (near the Americus eDNA collection site) was used to detect tagged fishes in this study.

### Collection of eggs

Prior to eDNA sample collection on a given date, we sampled for drifting eggs using a bongo net from either the bow of a boat or by wading into the river. Our sampling approach depended upon river discharge levels, with samples collected by wading under lower discharge conditions when boats could not be used. We used a paired bongo net (60‐cm‐diameter mouth, 333‐and 500‐μm bucket mesh sizes) positioned in the river's thalweg, just below the water surface because on many occasions water depths were similar to the height of the bongo net [Similar to Coulter *et al*. (2013) and Lenaerts *et al*. (2015)]. When deployed by wading, the net was held from each side, so field personnel did not interfere with the water flow through the net. Three bongo net samples were collected per site during each sampling event. The volume of water passing through a bongo net was determined by a flowmeter (G.O. Environmental; Miami, FL, USA) that was installed just inside the net's mouth. Collected samples were rinsed into white trays and bigheaded carp eggs were visually identified and counted using modified transfer pipets while in the field or were returned to the laboratory and counted. Egg counts were divided by water volumes to calculate egg density (number of eggs/m^3^), and the values for the three samples collected at each site were averaged. On Julian day 155, only one bongo net pull was completed because there was an unusually high number of eggs present in the drift. Drift samples were collected weekly and bigheaded carp eggs present were enumerated at Mascouten as part of an ongoing monitoring effort (Coulter 2015) and at all eDNA sites concurrent with water sample collection.

### Collection of water samples for eDNA extraction

We collected daily water samples between Julian days 149 and 173 during the 2013 field season (29 May and 22 June 2013), unless river conditions prevented safe collection of samples (i.e. flood events). We sampled 18 points that were evenly spaced along transects at each site (Mascouten, Americus and French Post). Transects were perpendicular to flow and spanned the wetted width of the river. The transect length (and sample point spacing) therefore varied based upon water levels. At each point, we collected a 50‐mL water sample by gently placing a Falcon conical tube (Fisher Scientific, Pittsburg, PA, USA) on the surface of the water column and allowing surface water and film to fill the tube to the 50‐mL mark (±5%). We changed gloves between each sample point to ensure sampling tubes would not be contaminated. We included field sample controls (i.e. tubes filled with reverse osmosis water in laboratory that were opened and closed in the field) to detect if field contamination occurred. After collection, we transported the samples back to Purdue University (West Lafayette, IN, USA) on ice for further processing.

### DNA extraction from the water sample

Upon returning to the laboratory, we centrifuged samples at 2440 ***g*** for 30 min (Merkes *et al*. 2014). We discarded the supernatant and collected the pellet using a sterile polyester swab. The cotton swabs were then frozen at −80 °C and shipped to USGS Upper Midwest Environmental Sciences Center for additional processing. We extracted DNA from the swabs using a commercial extraction kit (DNeasy Blood & Tissue; Qiagen Inc., Valencia, CA, USA) following the manufacturer's instructions with a final elution volume of 100 μL.

### qPCR amplification and eDNA quantification

We used a previously published method (Merkes *et al*. 2014) to conduct a quantitative polymerase chain reaction (qPCR) using bigheaded carp‐specific primers (forward‐GGTGGCGCAGAATGAACTA; reverse‐TCACATCATTTAACCAGATGCC) and a bigheaded carp‐specific taqman probe (sequence‐6‐FAM‐ CCATGTCCGTGAGATTCCAAGCC‐TAMRA). The probe anneals to bighead and silver carp sequences indiscriminately, but does not anneal to other Asian carp species. This marker was designed within the D‐loop region (GenBank: AB595924.1) of the mitochondrial genome. The qPCR cycling conditions were as follows: 95 °C for 2 min followed by 45 cycles of 95 °C for 10 s, 58 °C for 15 s, 61 °C for 15 s and a final extension at 61 °C for 5 min before holding at 4 °C. Reactions had a final volume of 20 μL and contained 1 μL of template DNA, 1‐μm forward and reverse primers, 50‐nm probe and 10 μL of 2× SensiFAST Probe No‐ROX Mix (Bioline USA Inc., Taunton, MA, USA). Each water sample was analysed in duplicate. Each plate had additional no template controls. We used a 7‐point calibration curve with plasmid DNA standards of 10^6^, 10^5^, 10^4^, 10^3^, 10^2^, 10 and 0 copies per reaction. The plasmid contained the cloned D‐loop region of silver carp (GenBank: AB595924.1). We calculated a standard curve using our standards: *copies *= 10^(Ct – 41.61)/−3.266)^ that had an *r* ^2^ of 0.864. All standards with a cycle threshold (Ct) >15 were used other than three points that were outliers based upon Grubb's test (Grubbs 1950). Amplification was detected by probe fluorescence at 520 nm, and DNA counts were calculated by mastercycler ep realplex software (version 2.2, Eppendorf North American, Hauppauge, NY, USA).

### Statistical analysis

Prior to analysis, we transformed the copy number with a log_10_ transformation to ensure normality. Copy numbers were then used to estimate the eDNA levels at each point along the transect. We visually examined the points along each transect by day and site to ensure no trends emerged across transects (Fig. S1, Supporting information). We then estimated the eDNA levels at each transect. The transect‐level estimates were then visually compared to discharge, egg densities and movement (telemetry) data. We only observed a relationship between movement data and eDNA copy numbers. We ultimately fit a hierarchical model to account for the different levels of observations (copy‐level data, points, transects and sites) (Gelman *et al*. 2013). Copy numbers were used to estimate the eDNA levels at each point (eqn 1). The estimates for each point were used to estimate the eDNA levels for each transect (eqn 2). The number of carp observed using telemetry data was used to predict the eDNA levels at the Americus transect after transformation (eqn 3).

(eqn 1) $$\log_{10}\left(\text{Copy Number}\right)∼\mathrm{Normal}\left(\mathrm{Point},\sigma_{\mathrm{point}}\right)$$

(eqn 2) $$\mathrm{Point}∼\mathrm{Normal}(\mathrm{Transect},\sigma_{\mathrm{transect}})$$

(eqn 3) $$\begin{array}{cc} & \mathrm{Transect}∼\mathrm{Normal}(\text{Telemetry Intercept}+\\ & \text{Telemetry Slope}\times \text{Telemetry count},\sigma_{\mathrm{telemery}}) \end{array}$$

All of these models were parameterized simultaneously using Stan (Homan & Gelman 2014) accessed via rstan in r (R Core Team 2015). We used four chains and 160 iterations (80 warm‐up and 80 sampling) to fit the model. We checked convergence using both the ^r diagnostic and by examining the traceplots (Gelman *et al*. 2013). We used the ggplot2 package within r to visualize data (Wickham 2009). A generalized linear model with Poisson error term was used to examine the relationship between discharge and the number of unique fish passing by Americus as detected by telemetry.

## Results

### Water discharge

Water discharge in the Wabash River study reach (French Post to Mascouten) varied considerably throughout the sampling period and among sites (Fig. S5, Supporting information). Spatially, Mascouten experienced much higher absolute discharges than Americus and French Post during the study period. Median discharges at the Lafayette gage ranged from 110 to 170 m^3^/s during the study period. Minimum discharges at Mascouten were higher than 230 m^3^/s during the study period, while both peak discharge rates occurred during rain events, resulting in discharges >850 m^3^/s. The first peak was on Julian day 154 with a mean daily discharge of 892 m^3^/s. The second peak was on Julian day 166 with a mean daily discharge of 954 m^3^/s. Gage heights during these peaks in discharge were approximately 5.2 m. Minor flood stage on the Wabash at Lafayette is 3.4 m while moderate flood stage is 6.1 m. Peak discharges during the study period at Mascouten were well beyond the minor flood stage.

Temporally, the sampling period spanned two large rain events, and bimodal rises in the hydrograph were observed at all three sampling sites. However, discharge levels did not consistently rise across the three sites. Mascouten had greater relative increases in discharge levels than either Americus or French Post, which is reasonable because a major tributary (the Tippecanoe River) and several minor tributaries that enter the Wabash River between the upper sites and Mascouten (Fig. 1). These observations suggest a heavier rain event affecting the drainage downstream of Americus during the first peak in discharge (i.e. Tippecanoe and Mascouten), whereas the second peak was relatively consistent across sites.

### Egg densities and telemetry

**Figure 2:**
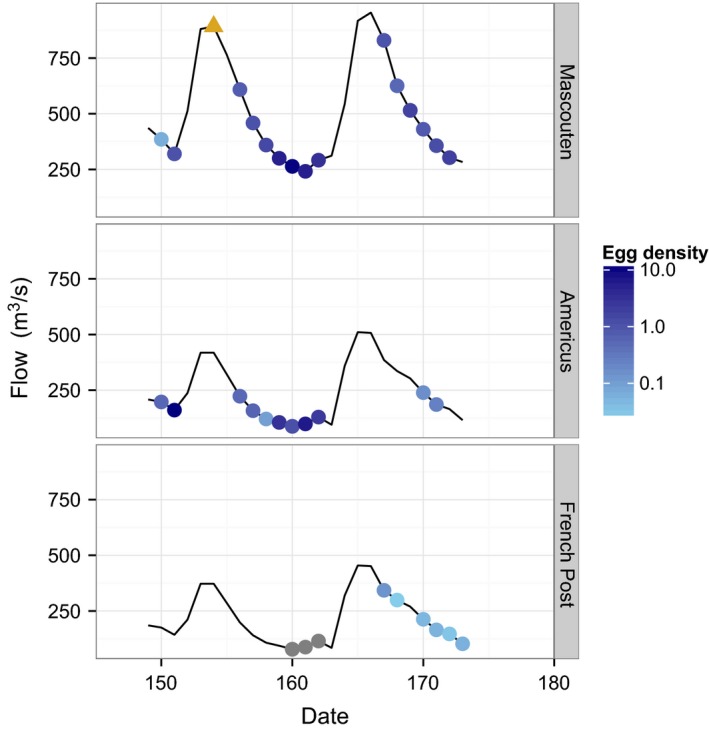
Discharge and egg densities (egg/m^3^). Discharge is the solid black line and egg densities are represented as shaded circles. The golden triangle is an outlier representing >10 000 eggs/m^3^ and was plotted separately to allow the other points to be differentiated. Grey points represent egg densities of zero.

Bigheaded carp eggs were observed at all three sites at varying densities (Fig. 2). The densities at Mascouten were highest following the first rise in the hydrograph. Observed egg density on Julian day 154 was >10 000 egg/m^3^, which was three orders of magnitude higher than the next highest observations at any site (~10 egg/m^3^). Egg densities were highest at Americus immediately before the first rise in hydrograph; however, samples were not taken at the peak of either hydrograph due to dangerously high water conditions. Egg densities at French Post were highest immediately following the second rise in the hydrograph. No eggs were observed at French Post prior to the second rise in the hydrograph. No trend emerged between discharge and egg density at any of the sites (Fig. S3, Supporting information).

**Figure 3:**
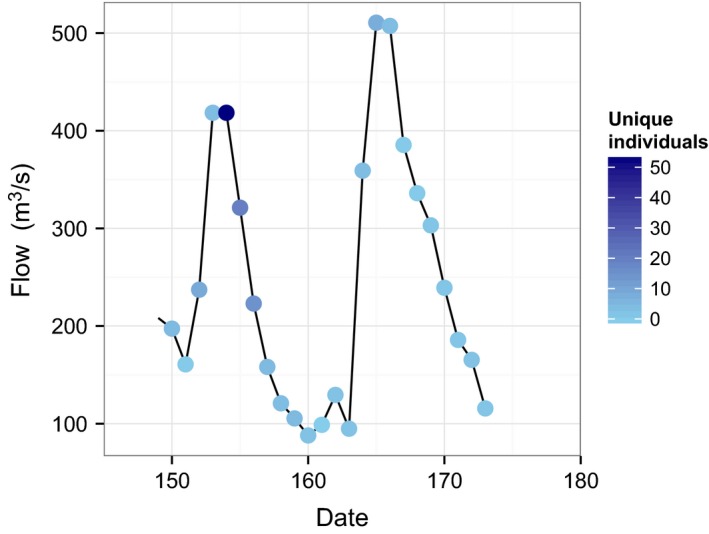
Daily flow data and unique fish detections at the Americus site. The shaded circles are daily unique detection counts.

The movement of bigheaded carps by Americus was greatest immediately after the first spike in the hydrograph (Fig. 3). More than 50 different tagged fishes were detected by the stationary receiver at Americus on Julian day 154. There was a slight increase in fish moving by Americus after the second spike in the hydrograph, but this spike was lower than the first spike in silver carp movement. In general, a weak trend emerged between discharge and the number of unique individuals crossing the Americus site [0.00373 carps/(m^3^/s); 95% credible interval: 0.00262–0.00485].

### eDNA

**Figure 4:**
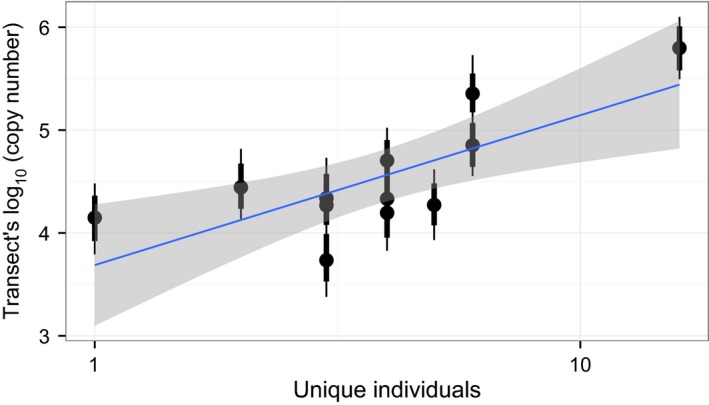
Relationship between telemetry data and the copy number of DNA observed at Americus. The blue line is from a linear regression and the shaded area is the 95% credibility interval. Black dots are the observed copy numbers and their 80% and 95% credibility intervals.

**Figure 5:**
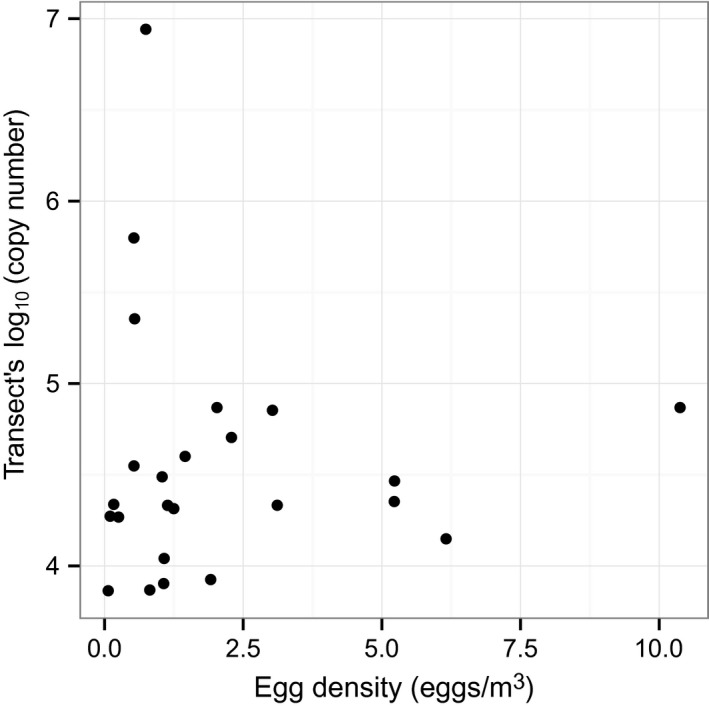
Relationship between eDNA copy number at each transect and the egg density at each transect.

We did not observe any consistent trends across eDNA sampling transects (e.g. high copy numbers from transect points in either the middle or edges of the river; Fig. S1, Supporting information). Copy numbers did vary through time and were highest after the first spike in the hydrograph at Americus and Mascouten (Fig. S2, Supporting information). Copy numbers were highest at Americus on the first observation day. Copy numbers were highest at French Post following the second rise in the hydrograph. We observed a positive slope, 0.961 (−0.402 to 2.52 95% credible interval), between observed telemetry counts and eDNA copy number (Fig. 4 and Table S1, Supporting information), but no relationship emerged between eDNA and egg density (Fig. 5).

## Discussion

Our results support using eDNA to monitor relative abundance (e.g. are there more or less present?) of species through time or space in addition to the presence/absence of species as part of a repeated sampling design. We found relationships among water discharge, eDNA and individual bigheaded carp movement that could be used to evaluate when spawning or other mass movements occur. We did not find a relationship between egg density and the other variables, which may be due to field sampling limitations or laboratory detection restrictions (e.g. not being able to sample during dangerous water conditions). Broadly, our findings agree with the known life history of bigheaded carp and the current limitations (e.g. DNA amplification, source uncertainty) of eDNA methods and our sampling approaches.

Linking eDNA to bigheaded carp movements allows monitoring to detect an important life history event: spawning. Bigheaded carps are known for having large‐scale movements close to spawning events (Abdusamadov 1987) that occur in the spring and early summer (Jennings 1988). These spawning events are driven by environmental factors, including changes in water levels (Jennings 1988; Peters *et al*. 2006; Duan *et al*. 2009). We observed an increase in tagged fish detections near Americus and eDNA copy numbers downstream of Americus associated with a rise in water level, which suggests a mass spawning event occurred upstream of the site. Our observation agreed with observations of bigheaded carps spawning upstream of Americus reported on the same day (154; Indiana Department of Natural Resources, unpublished data). The ~50 tagged bigheaded carps of the 300 tagged fish moving by the Americus stationary receiver suggested that large numbers of carps were moving upstream past this location, as tagged individuals represent a small portion of the Wabash River bigheaded carp population. Shortly after this large‐scale movement, egg densities >10 000 eggs/m^3^ were observed within 24 h of the mass movement at Mascouten. The eggs likely came from the large number of fish that spawned above Americus, and the eggs drifted downstream during development. Changes in water levels and velocities have been previously linked with movements of bigheaded carps (Calkins *et al*., 2012; DeGrandchamp *et al*. 2007) and other species (Lucas and Batley, 1996; Manion, 1977; Reynolds, 1983; Taylor and Cooke, 2012). Therefore, rises in hydrograph could be used to trigger eDNA sample collection and increase the likelihood of detecting an invasive species that moves in response to change in the hydrograph.

Additional spawning cues such as temperature, photoperiod and growing degree day can also influence when spawning and associated spawning movements may occur (Abdusamadov 1987; Kocovsky *et al*., 2012). While not examined in this study, these factors could add additional variation to our observations. Combined with our existing knowledge of bigheaded carp spawning triggers, eDNA could be used to detect fish movements and their approximate destination. Thus, our data suggest that eDNA may be a useful tool in monitoring movements of invading fishes by comparing the trends in detections and copy numbers over time. Single sampling events only provide a snapshot of the information, and by sampling consistently, additional information can be inferred from eDNA data that may be very useful to manage the resource.

We did not observe a relationship between eDNA and egg densities. We initially thought the lack of detection occurred because intact eggs did not release DNA. However, pilot laboratory studies indicated that intact eggs released large quantities of DNA under laboratory conditions that may or may not accurately represent field conditions (e.g. clean water without inhibitors present, no carps present that would be shedding DNA; S. McCalla, unpublished data). High amounts of variability also exist in the amount of DNA released from individual fish (Klymus *et al*. 2015); eggs may also exhibit similar variation in DNA shedding rates. This led us to two other possible hypotheses. First, we may have issues with amplification of the DNA in the samples. Inhibitors such as humic acid and algae present in the water sample may prevent amplification even if eDNA from the target species is present. Also, high levels of eDNA may oversaturate the methods used (Amberg *et al*. 2013). Second, we may have not sampled the water when eDNA was present from eggs. Our sampling was limited by high water, and we were unable to sample immediately after the peak discharge and we simply may have missed collecting water within the plume of eDNA presumably associated from the spawning event. Additionally, a sampling interval of 1 d may have been too short to capture fluctuations in egg density. Carp spawns can exhibit very strong pulse dynamics with large fluctuations occurring over the matter of minutes (D.C. Chapman, personal communication). Last, no method exists to differentiate DNA released from adults and eggs. If adults produce much greater quantities of eDNA, they may have overwhelmed any difference in signal produced by drifting eggs in our study.

The application of eDNA to aquatic environments is still a new technology that requires method refinement and development (Goldberg *et al*. 2015; Klymus *et al*. 2015). Our research highlights the needs for additional research regarding eDNA and spawning. First, additional studies are needed to quantify how eDNA detections vary by egg density and other sources of DNA during spawning (e.g. shed epithelial cells, sex products), to optimize approaches to address inhibition and to better understand degradation of DNA. Also, timing and frequency of field sampling requires additional refinement. We were unable to sample during the highest discharge conditions due to safety concerns, but the rising limb of the hydrograph may have had the highest spawning activity and egg densities (Yi *et al*. 1988; Zhang *et al*. 2000; Li *et al*. 2006). Conducting a study to address temporal variation with a greater frequency of sampling would provide insight into the diurnal variability of eDNA. Another limitation of our study and eDNA is that the one large fish may shed as much eDNA as many smaller fish (Klymus *et al*. 2015). Therefore, only inferences about the relative abundance of a species present through time or space may be drawn from eDNA data (e.g, there are likely more fish (or at least fish biomass) at Site A compared to Site B) rather than an absolute numeric comparison (e.g. Site A had X number of carp). Last, adapting and applying occupancy models [which are specific applications of finite mixture models (Gelman *et al*. 2013)] for eDNA would improve inferences drawn from these presence/absence data (Royle 2006; Royle & Link 2006; Royle & Kéry 2007).Our methods accounted for the nested transect design, but did not account for false negatives (carps present, but eDNA not detected) or false positives (eDNA detected, but carps not present). We also did not consider how many points or samples should be observed at a given transect from a statistical design. Our 18 points per transect are likely more than necessary. Optimizing sampling for eDNA requires active research and presents many open research questions on sampling design.

J.J.A., R.R.G. are conceived and designed the experiments. C.B.R., C.M.M., S.G.M., A.A.C., K.F.T., L.R.W. are performed the experiments. R.A.E., C.B.R., C.M.M., A.A.C., L.R.W. are analysed the data. J.J.A., R.R.G. are contributed reagents/materials/analysis tools. R.A.E., A.A.C., C.B.R., C.M.M. all wrote the study.

## Data Accessibility

Data use for this article is available at https://www.sciencebase.gov/catalog/item/57026390e4b0328dcb810247

## Supporting information


**Fig. S1** Observed copy numbers along sampling transects.
**Fig. S2** Observed copy numbers and flow through time at the three study sites.
**Fig. S3** Flow vs. egg density for all three sites.
**Fig. S4** Relationship between flow and the count of unique carp passing the Americus study site each day.
**Fig. S5** Wabash River discharge data for 2013 at the three transect locations (French Post, Americus, and Mascouten) and one major tributary (Tippecanoe's confluence, which is located immediately downriver from Americus).
**Table S1** Posterior distributions for regression parameters from the multilevel model used to examine the relationship between eDNA copy number and telemetry
