# Supplementary material for: Detecting the movement and spawning activity of bigheaded carps with environmental DNA
Source: Mol Ecol Resour. 2016 May 9;16(4):957–65. doi: 10.1111/1755-0998.12533 (PMC6680351; doi:10.1111/1755-0998.12533)
Supplement: Supplementary file 1 — Fig. S1 Observed copy numbers along sampling transects. Fig. S2 Observed copy numbers and flow through time at the three study sites. Fig. S3 Flow vs. egg density for all three sites. Fig. S4 Relationship between flow and the count of unique carp passing the Americus study site each day. Fig. S5 Wabash River discharge data for 2013 at the three transect locations (French Post, Americus, and Mascouten) and one major tributary (Tippecanoe's confluence, which is located immediately downriver from Americus). Table S1 Posterior distributions for regression parameters from the multilevel model used to examine the relationship between eDNA copy number and telemetry [file MEN-16-957-s001.pdf]

Detecting the movement and spawning activity of bigheaded carps with environmental DNA

Richard A. Erickson1*¶, Christopher B. Rees1¶§, Alison A. Coulter2¶†, Christopher M. Merkes1, S. Grace McCalla1¶, Katherine F. Touzinsky2, Liza Walleser1#, Reuben R. Goforth2&, Jon J. Amberg1&

Supplemental materials

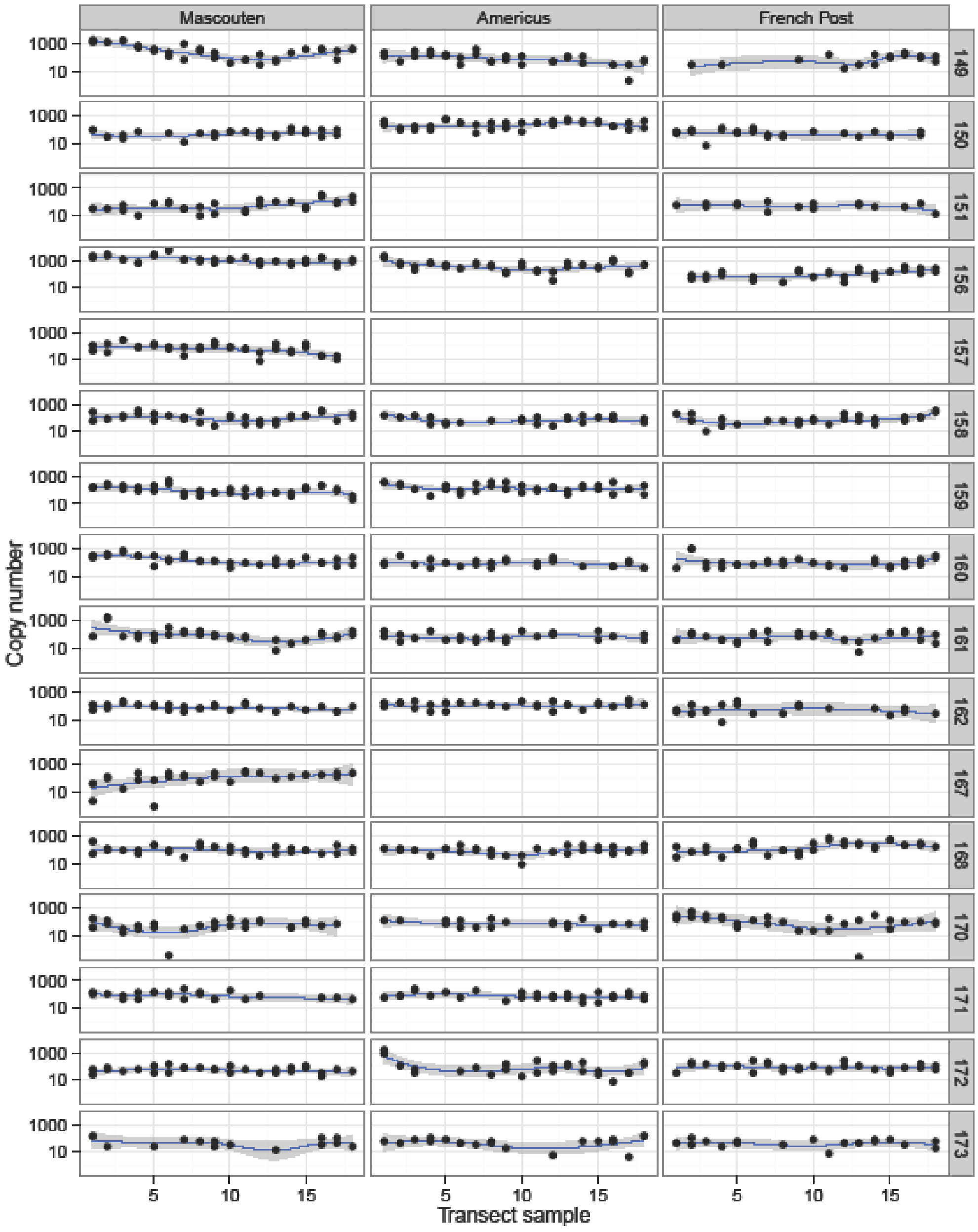

Supplemental Figure 1: Observed copy numbers along sampling transects. The blue line is a spline fitting to the data to help visualize any trends across transects. The figure is faceted by site on the x axis and sample day on the y-axis.

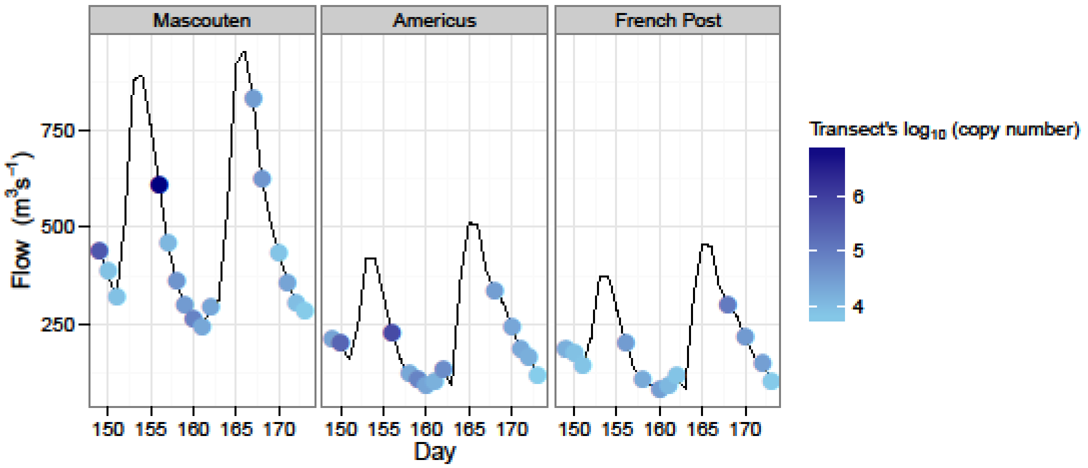

Supplemental Figure 2: Observed copy numbers and flow through time at the three study sites.

The dots are shaded based upon copy number at each transect.

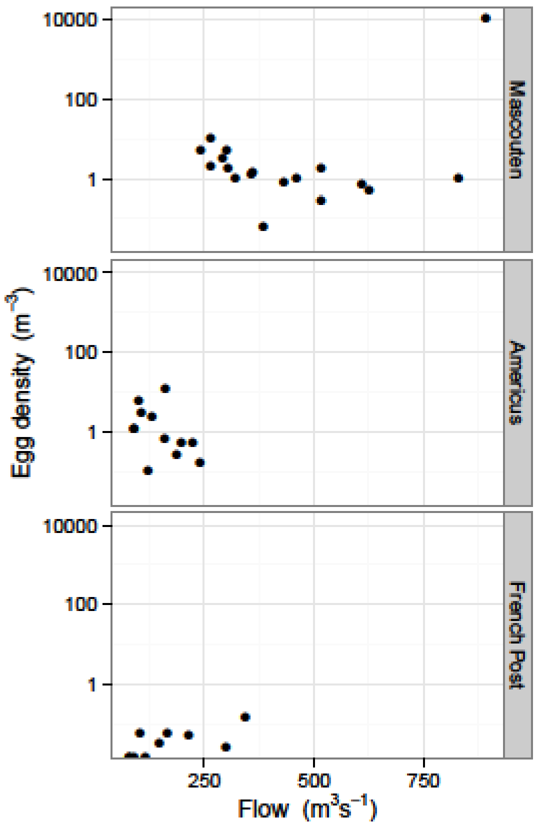

Supplemental Figure 3: Flow versus egg density for all three sites.

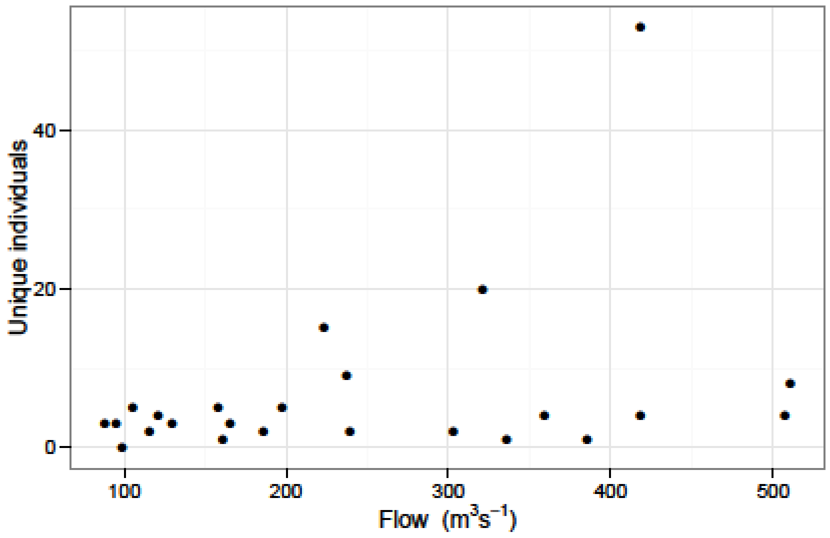

Supplemental Figure 4: Relationship between flow and the count of unique carp passing the Americus study site each day.

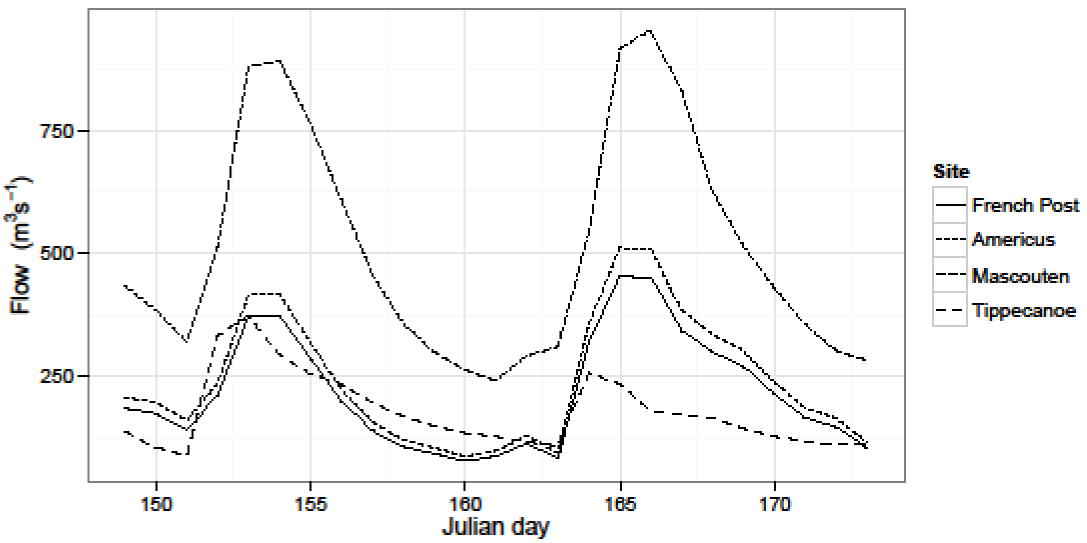

Supplemental Figure 5: Wabash River discharge data for 2013 at the three transect locations (French Post, Americus, and Mascouten) and one major tributary (Tippecanoe's confluence, which is located immediately downriver from Americus). Discharge rates at Mascouten were from the U.S. Geological Survey Surface-Water Data for the Nation database (U.S. Geological Survey) for the Wabash River at Lafayette, IN (site number 03335500). Discharge rates for Americus and French Post were calculated by the USGS Illinois Water Sciences Center using the drainage area ratio method with data obtained from the U.S. Geological Survey stream gage at Logansport, IN (site number 03329000).

Supplemental Table 1: Posterior distributions for regression parameters from the multilevel model used to examine the relationship between eDNA copy number and telemetry

	2.5%	10%	50%	90%	97.5%
sigmaPoint	0.5461674	0.5563709	0.5812875	0.6050475	0.6172601
sigmaTransect	0.4565160	0.4783862	0.5164018	0.5508169	0.5703470
sigmaTel	3.2159583	3.4748347	3.8931717	4.4943849	4.8455869
tel0	1.2857339	1.7847703	3.0759970	4.3142257	4.7489119
tel1	-0.4024899	0.0884802	0.9651263	2.0660596	2.5222389
